# Immunological tumor status may predict response to neoadjuvant chemotherapy and outcome after radical cystectomy in bladder cancer

**DOI:** 10.1038/s41598-017-12892-5

**Published:** 2017-10-04

**Authors:** Minna Tervahartiala, Pekka Taimen, Tuomas Mirtti, Ilmari Koskinen, Thorsten Ecke, Sirpa Jalkanen, Peter J. Boström

**Affiliations:** 1 MediCity Research Laboratory, Department of Medical Microbiology and Immunology, University of Turku, Turku, Finland; 2 Department of Pathology, University of Turku and Turku University Hospital, Turku, Finland; 3 Helsinki University Hospital, Department of Pathology (HUSLAB) and Medicum, University of Helsinki, Helsinki, Finland; 4 Department of Urology, Helsinki University Hospital and University of Helsinki, Helsinki, Finland; 5 Department of Urology, HELIOS Hospital Bad Saarow, Bad Saarow, Germany; 6 Department of Urology, Turku University Hospital, Turku, Finland

## Abstract

Bladder cancer (BC) is the ninth most common cancer worldwide. Radical cystectomy (RC) with neoadjuvant chemotherapy (NAC) is recommended for muscle-invasive BC. The challenge of the neoadjuvant approach relates to challenges in selection of patients to chemotherapy that are likely to respond to the treatment. To date, there are no validated molecular markers or baseline clinical characteristics to identify these patients. Different inflammatory markers, including tumor associated macrophages with their plastic pro-tumorigenic and anti-tumorigenic functions, have extensively been under interests as potential prognostic and predictive biomarkers in different cancer types. In this immunohistochemical study we evaluated the predictive roles of three immunological markers, CD68, MAC387, and CLEVER-1, in response to NAC and outcome of BC. 41% of the patients had a complete response (pT0N0) to NAC. Basic clinicopathological variables did not predict response to NAC. In contrast, MAC387^+^ cells and CLEVER-1^+^ macrophages associated with poor NAC response, while CLEVER-1^+^ vessels associated with more favourable response to NAC. Higher counts of CLEVER-1^+^ macrophages associated with poorer overall survival and CD68^+^ macrophages seem to have an independent prognostic value in BC patients treated with NAC. Our findings point out that CD68, MAC387, and CLEVER-1 may be useful prognostic and predictive markers in BC.

## Introduction

Bladder cancer (BC) is the fourth most common cancer in men in developed countries. In 2012 429,800 new cases were recorded and 165,100 deaths occurred worldwide due to BC^1^. Radical cystectomy (RC) with cisplatin-based neoadjuvant chemotherapy (NAC) prior to the surgery is recommended for muscle-invasive BC. However, NAC is only effective in 30–40% of patients, and there are no validated molecular markers or baseline clinical characteristics to adequately identify the patients who are likely to benefit from the treatment^2,3^. Patients with no response to NAC are subject to adverse effects of chemotherapy and delay in the definitive treatment. Hence, there is an urgent need for new biomarkers to guide therapeutic decisions in the treatment of BC.

Different inflammatory markers have been extensively investigated as potential prognostic and predictive biomarkers^4^. Tumor associated macrophages (TAMs) are attractive targets as biological markers, as well as in therapeutic strategies, with their plastic pro-tumorigenic and anti-tumorigenic functions^5,6^. The presence of TAMs in solid tumors favors tumor growth and progression^7^. Few studies have shown the capability of chemotherapy treatment to switch the TAM polarization from protumoral into more M1-like phenotype with tumoricidal and proinflammatory functions^8–10^. However in BC, the association of TAMs and other potential immunological markers with NAC is unknown.

In our previous study, we showed that CD68^+^ and MAC387^+^ macrophages associate with conventional high-risk features in BC, the risk of progression and poorer survival in BC patients^11^. We also showed that, by contrast, CLEVER-1^+^ vessels associate with lower risk for progression. In the present study the objective was to evaluate the roles of these three immunological markers (CD68, MAC387, and CLEVER-1) and NAC response in BC. CD68 is the most frequently used pan-macrophage marker, while MAC387 is expressed on recently infiltrating monocytes/macrophages and is considered as a marker of active inflammation^12^. In addition, various tumor cells also express MAC387^13^. We have previously shown MAC387 being expressed by BC tumor cells^11^. CLEVER-1 is an immunosuppressive scavenger receptor expressed by lymphatic and vascular endothelial cells and tissue macrophages^14^. To our best knowledge, MAC387 and CLEVER-1 have not been evaluated as biological markers for NAC response. The density of CD68^+^ macrophages has been shown to associate with chemoresponse in pancreatic, breast and lung cancer^8,15,16^, but the relationship between NAC response and CD68^+^ macrophages in BC has not been studied.

## Results

### Clinicopathological characteristics

The baseline clinicopathological characteristics of the patient cohort were evaluated and they are presented in Table 1. All the patients (n = 68) had urothelial BCs and received NAC. Six patients received adjuvant chemotherapy after the RC. The cohort is a typical RC cohort.

**Table 1:** Baseline clinicopathological characteristics, n = 68.

Characteristic, n = 68		n (%)
Age (years)	Mean/median (range)	64/65 (47–76)
Gender	Male	58 (85)
Smoking (current or past)	Yes	52 (76)
cT category (TUR-BT’)	T2	48 (71)
	T3	18 (27)
	T4	2 (3)
CIS^1^ (TUR-BT’)	Yes	15 (22)
LVI^2^ (TUR-BT’)	Yes	17 (25)
Tumor size (mm) (TUR-BT’)	Mean/median (range)	23/19 (1–70)
pT category (RC”)	T0	28 (41)
	pTa, pTcis, pT1	15 (22)
	pT2	10 (15)
	pT3	10 (15)
	pT4	5 (7)
pN category (RC”)	Positive	7 (11)
	Negative	58 (89)
Neoadjuvant chemotherapy	Cisplatin-Gemcitabine	64 (94)
	Carboplatin-Gemcitabine	4 (6)
Chemotherapy cycles (number)	Mean/median (range)	3/4 (2–6)^3^
Adjuvant chemotherapy	Yes	6 (9)
Follow-up time (years)	Mean/median (range)	3.3/3.6 (0.25–7.7)
Status	Alive, no evidence of disease	53 (78)
	Death due bladder cancer	13 (19)
	Death due other reason	2 (3)
Pathological response to the neoadjuvant chemotherapy	Complete response (pT0)	28 (41)
	Partial response (pT1/pTa/pTis)	14 (21)
	No response	9 (13)
	Progression (pT3 and/or N+)	17 (25)

’According to the TUR-BT pathology, imaging studies and clinical status.

”According to the pathological data from RC.

^1^Concomitant carcinoma *in situ*.

^2^Lymphovascular invasion.

^3^9 patients (13%) received 2 cycles of NAC, 23 patients (34%) 3 cycles, 34 patients (50%) 4 cycles, and 1 patient 6 cycles.

### Manual macrophage counting correlates with digital counting

**Figure 1:**
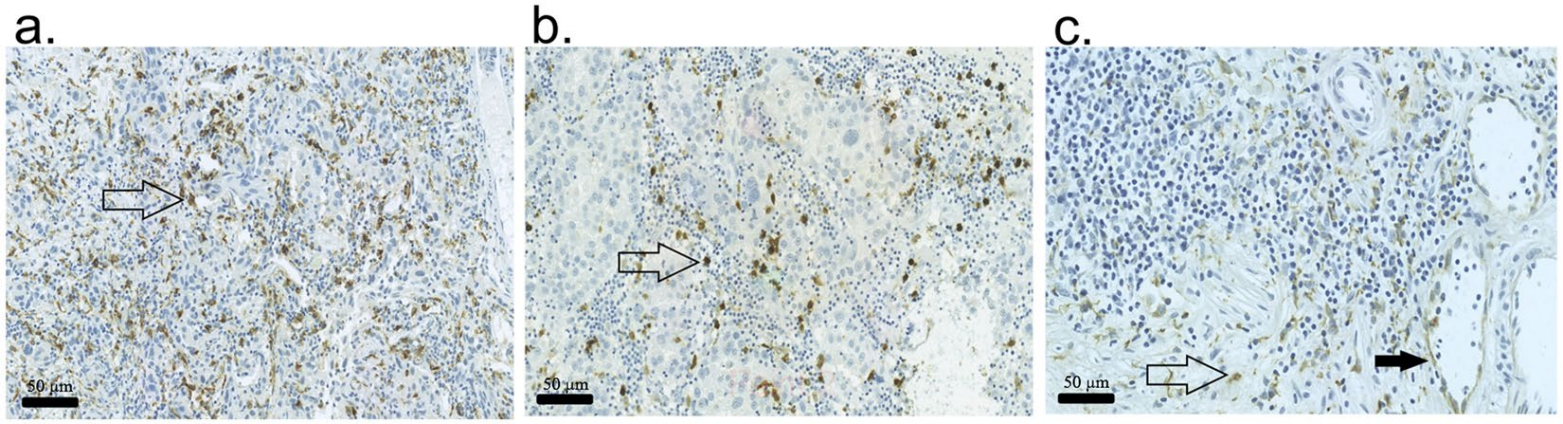
Representative examples showing immunohistochemical stainings of studied markers in TUR-BT specimens. Examples of (**a**) CD68, (**b**) MAC387, and (**c**) CLEVER-1 staining patterns. ⇧ indicates positively stained macrophages,  indicates a positive CLEVER-1 vessel.

Histological samples from 68 NAC and RC treated BC patients were stained with CD68, MAC387 and CLEVER-1 primary antibodies. Positive cells and vessels were counted manually from TUR-BT (transurethral resection of bladder tumor) sections. TMAs (tissue microarray) were created and the positive cells were counted manually to evaluate the use of TMA in the study of macrophages. Positive cell counts were dichotomized according to the mean value and the groups from manually counted whole sections are shown in Table 2. TUR-BT sections were also counted digitally to study different techniques when analysing immunohistochemical samples. Marker counts from manually counted TMA samples and digitally counted TUR-BT whole section samples are shown in Supplementary Table S1. Representative examples of the staining patterns with different markers are represented in Fig. 1.

**Table 2:** Marker counts from TUR-BT sections.

Marker		n (%)
CD68 (n = 64)	Low (0–59)	30 (44)
	High (60–175)	38 (56)
MAC387 (n = 62)	Low (8–78)	37 (54)
	High (79–240)	31 (46)
MAC387 tumor^1^ (n = 66)	0	9 (14)
	1	29 (44)
	2	15 (23)
	3	12 (18)
CLEVER-1m^2^ (n = 65)	Low (0–53)	34 (50)
	High (54–209)	34 (50)
CLEVER-1v^3^ (n = 66)	Low (0–4)	39 (57)
	High (5–27)	29 (43)

Groups dichotomized according to the mean value.

^1^MAC387 positive tumor cells, semiquantitative scoring.

^2^CLEVER-1 positive macrophages.

^3^CLEVER-1 positive blood and lymph vessels.

Manual and digital counts correlated with all markers (CD68 p = 0.008, MAC387 p < 0.001, and CLEVER-1 p < 0.001) (Supplementary Table S2). CD68^+^ and MAC387^+^ macrophage counts from TMAs (tissue microarray) and whole sections correlated with each other (p = 0.002 for CD68 and p < 0.001 for MAC387, respectively), but CLEVER-1^+^ macrophages or vessels did not (both p = 0.41) (Supplementary Table S2). All of the correlation and survival analyses were performed with the results from the TUR-BT whole sections.

### High CD68^+^ macrophage count associates with LVI

**Figure 2:**
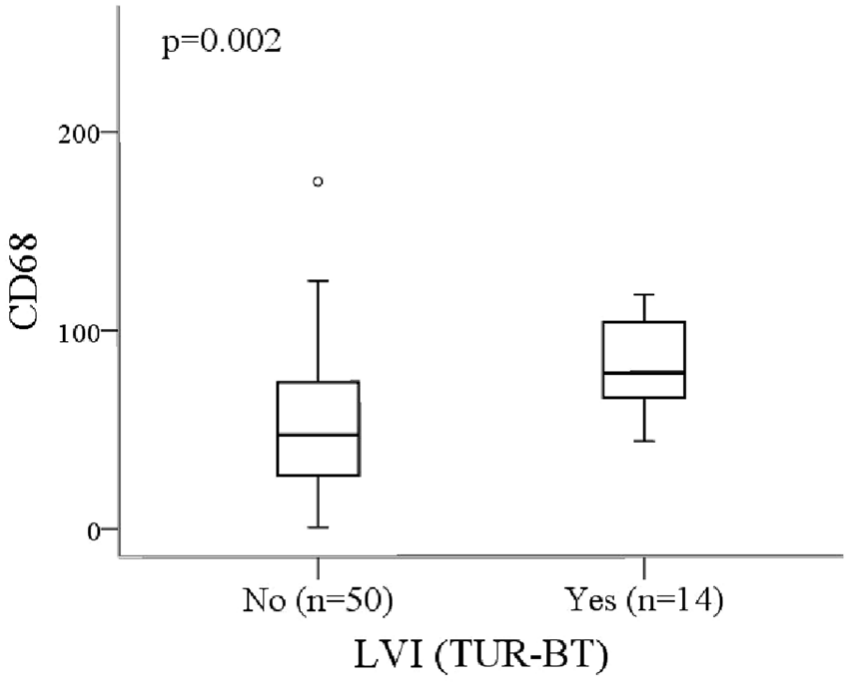
High CD68^+^ macrophage counts associate with the presence of LVI (Mann-Whitney U test).

Associations between immunohistological markers and clinicopathological variables were analysed. High CD68^+^ macrophage count correlated with the presence of lymphovascular invasion (LVI) (p = 0.002, Fig. 2). MAC387^+^ tumor cells or CLEVER-1^+^ macrophages/vessels did not associate with LVI (Supplementary Table S3). There were no associations noticed between immunohistological markers and other clinicopathological variables (Supplementary Table S3).

### MAC387 and CLEVER-1 stainings identify chemosensitive/chemoresistant tumors

**Figure 3:**
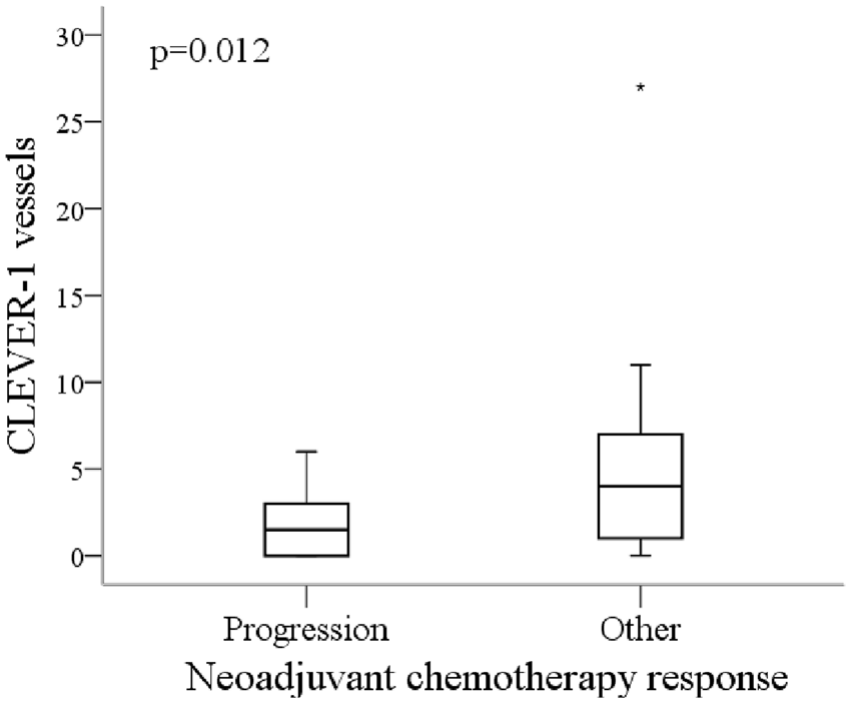
Association between neoadjuvant chemotherapy response (progression and other response) and CLEVER-1^+^ vessels (Mann-Whitney U test).

The association between the chemotherapy response and markers studied, and the chemotherapy response and the clinicopathological characteristics were analysed. There were no associations between response to NAC and clinicopathological characteristics (gender, smoking, type of chemotherapy, tumor grade, or presence of CIS or LVI) (Supplementary Table S4). Associations between markers and chemotherapy response are shown in Table 3. CD68^+^ and MAC387^+^ macrophages did not associate with the chemotherapy response. However, higher MAC387^+^ tumor cell density (scoring 0–2 vs. 3) associated with the risk of progression following NAC (progression vs. other response; hazard ratio (HR) 3.76, 95% confidence interval (CI) 1.10–12.82, p = 0.034). 47% and 19% of the patients with high and low amounts of MAC387^+^ tumor cells progressed during NAC, respectively. On the other hand, 47% of the patients with low amount of MAC387^+^ tumor cells had a complete NAC response compared to 20% in high number group (Supplementary Table S5). High CLEVER-1^+^ macrophage count associated significantly with poorer response to NAC (complete/partial response vs. no response/progression; HR 2.78, 95% CI 1.00–7.67, p = 0.049). In contrast, a significant association between a lower count of CLEVER-1^+^ vessels and progression during NAC was noticed (Mann-Whitney U-test p = 0.012) (Fig. 3).

**Table 3:** Associations between markers and chemotherapy response.

Variable		Complete *vs*. other			Complete/Partial *vs*. other			Other *vs*. progression		
		HR	95% CI	p-value	HR	95% CI	p-value	HR	95% CI	p-value
CD68	Low *vs*. high	0.72	0.27–1.91	0.50	1.13	0.42–3.023	0.81	1.63	0.52–5.078	0.40
	Continuous			0.33			0.58			0.97
MAC387	Low *vs*. high	0.94	0.36–2.49	0.91	1.038	0.39–2.77	0.94	1.48	0.49–4.46	0.48
	Continuous			0.42			0.40			0.53
MAC387tumor^1^	Low *vs*. high	3.57	0.90–14.13	0.070*	3.18	0.97–10.37	0.056*	3.76	1.10–12.82	0.034*
	All groups 0–3			0.41			0.36			0.40
CLEVER-1m^2^	Low *vs*. high	1.63	0.62–4.32	0.33	2.78	1.006–7.67	0.049*	1.61	0.53–4.88	0.40
	Continuous			0.49			0.21			0.88
CLEVER-1v^3^	Low *vs*. high	0.99	0.37–2.62	0.98	0.76	0.28–2.050	0.58	0.47	0.14–1.52	0.21
	Continuous			0.26			0.10			0.012*

Regression analyses were used to evaluate the association between the chemotherapy response and marker groups dichotomized according to the mean value. Mann-Whitney U test was used to evaluate the association between the continuous variables and chemotherapy response. Pearson Chi-square/Fisher’s exact test was used to evaluate the association between MAC387^+^ tumor cells and chemotherapy response.

^1^MAC387 positive tumor cells; low (score 0–2), high (score 3).

^2^CLEVER-1 positive macrophages.

^3^CLEVER-1 positive vessels.

^*^Significant p-value.

### High macrophage counts associate with poorer survival

**Figure 4:**
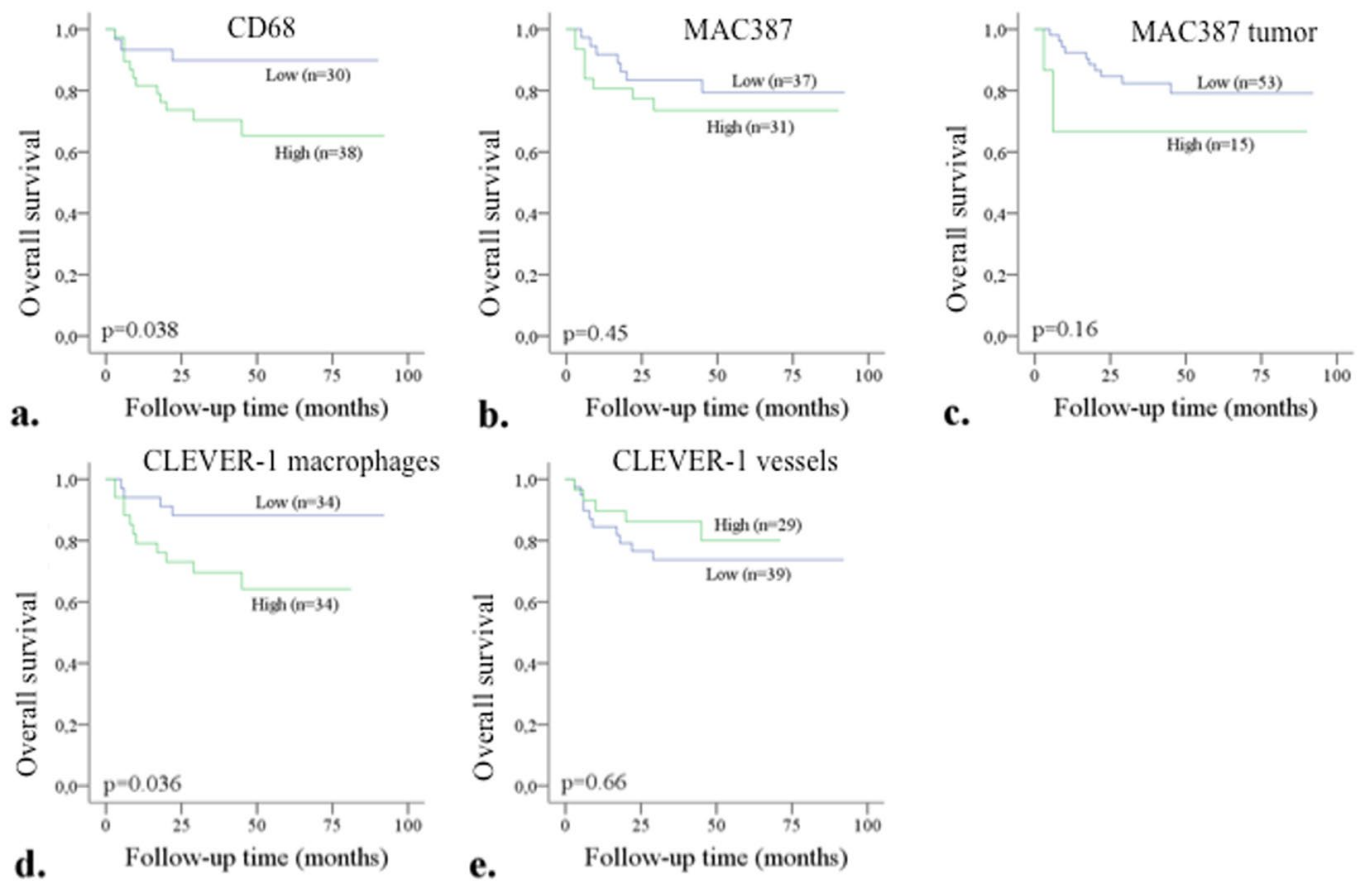
Kaplan-Meier estimates for OS after NAC treatment. The effect of CD68^+^ (**a**), MAC387^+^ macrophages (**b**), MAC387^+^ tumor cells (**c**), CLEVER-1^+^ macrophages (**d**) and CLEVER-1^+^ vessels on the OS in TUR-BT specimens in BC patients receiving NAC. The markers were dichotomized into two groups according to the mean value.

Survival analyses were performed with Kaplan-Meier estimates and uni- and multivariate Cox proportional hazard regression models. Kaplan-Meier estimates evaluating the relationships between the markers and OS (overall survival) are shown in Fig. 4. High CD68^+^ and CLEVER-1^+^ macrophage counts associated with poorer OS after NAC and RC (p = 0.038 for CD68, and p = 0.036 for CLEVER-1, respectively). MAC387^+^ macrophage/tumor cell counts and CLEVER-1^+^ vessels did not associate with survival.

Univariate and multivariate Cox proportional hazard regression models of factors affecting OS are presented in Table 4. In the univariate analyses, fewer chemotherapy cycles, adjuvant chemotherapy, the presence of LVI in TUR-BT specimens, lymph node positivity, and tumor in RC (*vs*. pT0) significantly associated with shorter OS. In addition, higher counts of CLEVER-1^+^ macrophages significantly associated with OS in univariate analysis (HR 3.17, 95% CI 1.01–9.97, p = 0.048), but failed to remain significant in multivariate analysis with pT-category (HR 2.94, 95% CI 0.93–9.27, p = 0.066). CD68^+^ macrophages, however, showed a significant association with OS in multivariate analysis after adjusting for pT-category (HR 3.97, 95% CI 1.11–14.12 p = 0.033).

**Table 4:** Univariate and multivariate Cox proportional hazards regression analysis of factors affecting OS.

OS		Univariate			Multivariate		
Variable		HR	95% CI	p-value	HR	95% CI	p-value
Age		1.009	0.939–1.083	0.81			
Gender	Male		*REF*				
	Female	2.43	0.77–7.63	0.13			
Smoking	No		*REF*				
	Yes	0.44	0.12–1.64	0.22			
Neoadjuvant chemotherapy	Cisplatin-Gemcitabine		*REF*				
	Other	1.00	0.13–7.59	1.00			
Chemotherapy cycles	0.50	0.28–0.91		0.022*			
Adjuvant chemotherapy	No		*REF*				
	Yes	4.32	1.37–13.60	0.012*			
LVI (TUR-BT’)	No		*REF*				
	Yes	3.072	1.11–8.51	0.031*			
pN category (RC”)	Negative		*REF*				
	Positive	5.36	1.82–15.82	0.002*			
pT category (RC”)	T0		*REF*			*REF*	
	Other	5.46	1.23–24.22	0.026*	4.60	1.004–21.022	0.049*
CD68 continuous		1.009	0.994–1.023	0.24	1.008	0.995–1.021	0.23
MAC387 continuous		1.002	0.990–1.014	0.80	1.002	0.991–1.013	0.73
CLEVER-1m^1^ continuous		0.999	0.981–1.018	0.95	0.998	0.982–1.015	0.82
CLEVER-1v^2^ continuous		0.873	0.718–1.062	0.17	0.901	0.748–1.086	0.28
CD68 dichotomized		3.50	0.99–12.44	0.053	3.97	1.11–14.12	0.033*
MAC387 dichotomized		1.48	0.54–4.08	0.45	1.57	0.57–4.32	0.39
MAC387tumor^2^ dichotomized		2.13	0.73–6.25	0.17	1.65	0.56–4.89	0.36
CLEVER-1m^1^ dichotomized		3.17	1.01–9.97	0.048*	2.94	0.93–9.27	0.066
CLEVER-1v^2^ dichotomized		0.64	0.22–1.87	0.42	0.65	0.22–1.90	0.43

The stage of the tumor was combined with each marker separately in multivariate analyses. ’According to the TUR-BT pathology, imaging studies and clinical status﻿﻿. ’’According to the pathological data from RC.

^1^CLEVER-1 positive macrophages.

^2^CLEVER-1 positive vessels.

^3^MAC387 positive tumor cells.

*Significant p-value.

## Discussion

In the present study, we have evaluated the role of three different immunological markers in BC patients treated with NAC. We demonstrated that high CD68^+^ and CLEVER-1^+^ macrophage counts associate with poorer OS after NAC and RC. Furthermore, high CD68^+^ macrophage count was an independent predictive factor for poor OS. When the response to NAC was analysed, there were no associations between the response and clinicopathological characteristics, but interestingly, MAC387^+^ tumor cell density associated with the response to NAC. High MAC387^+^ tumor cell density associated with disease progression during NAC, whereas the majority of the patients with lower amount of MAC387^+^ tumor cells received a complete response. CLEVER-1^+^ macrophage and vessel counts associated significantly with response to NAC. Patients with high amounts of CLEVER-1^+^ macrophages had poorer response to NAC, while higher amounts of CLEVER-1^+^ vessels associated with more favourable response.

To date, there are no validated biomarkers in clinical use to predict the outcome of NAC among BC patients. Some of the most interesting markers include e.g. immunohistochemical evaluation of Emmprin as demonstrated by Hemdan *et al*.^17^. In 2014, Choi *et al*. identified basal and luminal subtypes of muscle-invasive BC and demonstrated that immune-infiltrated basal BCs respond to NAC and should be managed aggressively with NAC to improve the survival of the patients^18^.

Immunological factors have an indisputable role in cancer development^19^. TAMs have a dual role in the tumor microenvironment; they are potentially tumoricidal but can also promote cancer cell proliferation^20,21^. TAMs have an essential role in different therapeutic strategies against cancer. Chemotherapy can inhibit or activate monocyte/macrophage mediated anti-tumor responses^22^ and the modulation of tumor responses to chemotherapy can vary between different cytotoxic factors and tumors^23^. Immunological factors and TAMs have not been studied thoroughly among chemotherapy treated BC patients. There are a few studies evaluating the role of peripheral blood lymphocytes in BC patients receiving NAC^24–26^, but there is a lack of studies evaluating the role of immunological cells in the tumor microenvironment. To our best knowledge, this is the first study to evaluate the relationship between NAC and immunological markers CD68, MAC387 and CLEVER-1 as predictive markers in BC patients. The results are in line with our previous study, where we demonstrated that CD68^+^ and MAC387^+^ macrophages associate with high risk factors and poorer survival in BC patients while CLEVER-1^+^ vessels predict more favourable outcome^11^. Although further validation studies are still needed, our results strongly suggest that immunological factors do play an important role in NAC response, and such markers could be used in clinical practice to identify patients who benefit from the treatment. Especially MAC387 could be used to identify the tumors that are more prone to progress during NAC (high MAC387^+^ cell count) or receive an expected response (low MAC387^+^ cell count).

Different therapeutic agents can influence plastic TAMs causing inhibition or activation on antitumor responses^22^. Mantovani *et al*. demonstrated in 2013, that tissue damage caused by chemotherapeutic treatments can lead to misdirected macrophage-orchestrated tissue repair response and promotion of tumor growth and limited antineoplastic efficacy^27^. Our results show, that the risk of progression after NAC increases when there is higher amounts of MAC387^+^ tumor cells. It could be speculated, that MAC387 introduces the misdirected tissue repair orchestrated by TAMs, and thus, restrain the effect of the treatment. In the other hand, we demonstrated that immunosuppressive CLEVER-1 enhanced the chemotherapy response. We have previously shown, that CLEVER-1^+^ vessels associate with improved survival in BC^11^, but however, this association with survival after NAC, could not be seen in the present study.

The present study has the known limitations of a retrospective study. The number of patients was limited, but contained consecutive BC patients receiving NAC prior to RC from two academic referral centers in Finland in 2008–2013. Macrophages are challenging to investigate with immunohistochemistry due to their nature to cluster. This may lead to variation in results especially when using TMAs and would require sufficient tissue sampling in routine clinical practice. Both the TMA and whole section based cell counting techniques were tested in the present study. The results from CD68 and MAC387 stainings correlated with each other, but the results from CLEVER-1 quantifications were different when using TMAs and whole sections. TMAs are an efficient method in immunohistochemistry, but it should be considered attentively when studying clustering particles, e.g. macrophages. We also compared manual and digital counting of macrophages. Manual counting is subjective and time-consuming but more accurate when sorting different cell types and artefacts, while digital counting is objective and repeatable, but vulnerable to artefacts such as diffusely distributed necrotic tissue material. In this study, however, we demonstrated that digital counting with Fiji-ImageJ is reliable enough to determine the number of macrophages in an automated fashion.

In conclusion, we found that MAC387^+^ cells and CLEVER-1^+^ macrophages and vessels associate with the response after NAC in BC patients. High MAC387^+^ tumor cell density associated with disease progression after NAC, whereas majority of patients with lower amount of MAC387^+^ tumor cells received a complete response. Patients with high amounts of CLEVER-1^+^ macrophages associated with poorer response to NAC, while higher amounts of CLEVER-1^+^ vessels associated with more favourable response. The results verify also our previous studies where we demonstrated that CD68 and MAC387 associate with poorer survival in BC patients whereas CLEVER-1 vessels act more as a protective marker. Further studies are needed to validate the results of immunological markers predicting NAC outcome in BC patients.

## Methods

### Patients

All BC patients undergoing NAC followed by RC from Helsinki (years 2010–2013) and Turku (2007–2013) University Hospitals were included in the study (n = 76). After exclusion of patients with non-urothelial histology, inadequate (<2 cycles) NAC and insufficient tissue material, 68 patients were included in the study.

TUR-BT was performed using standard technique. Patients received NAC, 2–6 cycles either cisplatin-gemcitabine (64/68 patients), or carboplatin-gemcitabine (4/68 patients) prior to the RC. RC included removal of the bladder, prostate, and seminal vesicle in men and the uterus, ovaries, and anterior vaginal wall in females. All patients had pelvic lymph node dissection (PLND). The PLND template was decided by responsible surgeon and the nodal specimens were evaluated according institutional pathology guidelines. 42/68 (62%) had an extended PLND and 26/68 (38%) limited dissection with definitions similar to the paper by Dhar *et al*.^28^. In the extended dissection, the upper limit of dissection was aortic bifurcation or ureteric crossing of the iliac vessel, and in limited the dissection was distal to iliac bifurcation. The mean number of the removed nodes in the whole cohort was 19 (24 in patients with extended dissection, 10 with limited).

A detailed database was collected retrospectively including detailed patient data and tumor characteristics, as well as details of the treatment and follow-up. Histological tissue samples were re-reviewed by two expert uro-pathologists in consensus (P.T., T.M.). The study protocol was approved by the Research Ethical Board of the Hospital District of Southwestern Finland. All methods were carried out in accordance with relevant guidelines and regulations. The study was conducted in compliance with the current revision of the Declaration of Helsinki guiding physicians and medical research involving human subjects. A written informed consent from the patients was obtained. The study did not affect the patients or there further treatment of follow-up in any way. All the sample collections were done on already existing tissue specimens received during the diagnosis and treatment of these patients.

### Immunohistochemistry and scoring

Formalin-fixed, paraffin-embedded tissue blocks were cut at three μm thickness. The detailed protocol of the immunohistochemistry has been previously reported^11^. The primary antibodies used were mouse monoclonal IgG_1_ anti-CD68 (concentration 1/5, KP1, ab845, Abcam, U.K.), mouse monoclonal IgG_1_ anti-MAC387 (concentration 1/500, ab22506, Abcam, U.K.) and rat IgG 2–7 (concentration 1/5) against CLEVER-1/Stabilin-1^29,30^. Mouse IgG_1_ 3G6^31^ and rat IgG_2a_ anti-mouse CD62L (MEL-14, Exbio, Czech Republic) were used as negative controls.

From TUR-BT samples, the whole sections of paraffin-embedded blocks were analysed as well as the TMAs (tissue microarray). The immunohistological stainings were analysed manually and digitally blinded to the clinical information. Manual analyses were performed microscopically from three hotspots using a 0.0625 mm^2^ grid with 20x (vessels) or 40x (macrophages) magnifications. The most macrophages/vessels containing hotspots were selected from the samples. The number of macrophages and vessels were counted within each hotspot and the mean numbers per field were calculated. MAC387^+^ tumor cells were graded semiquantitatively into four categories 0–3 (from none to abundant). For digital analyses, sections were scanned with Pannoramic 250 Slide Scanner (3DHISTEC). Three hotspots were chosen from the scanned images and analysed with Fiji-ImageJ 2.0.0. Shortly, the macrophage-positive areas were extracted by colour deconvolution and the resulting image was thresholded. Then a size limit was applied and macrophage-positive areas were calculated. For MAC387 analyses, the images were watershed and a size limit was applied to exclude the larger positive tumor cells from macrophages. The mean percentages of the hotspots were calculated and used in analyses. CLEVER-1^+^ vessels were analysed manually.

### Data availability

The datasets generated and analysed during the current study are available from the corresponding author on reasonable request.

### Statistical Analyses

Spearman rank-order correlation coefficient was used to test the correlations between manual *vs*. digital cell counting from whole sections and TMA *vs*. whole sections. Associations between clinicopathological characteristics and markers were evaluated with Spearman rank-order correlation coefficient, Mann-Whitney U test and Kruskal-Wallis test. NAC response was categorized as follows: complete response (pT0N0), partial response (pT1/pTa/pTisN0), no response (pT2N0), and progression (pT3 and/or N+). Associations between the chemotherapy response and clinicopathological characteristics were evaluated with Pearson Chi-square. Fisher’s exact test was used when >20% of the cells had expected count less than five or the minimum expected count was <1. Analysed macrophage and vessel markers were dichotomized by the mean value (low *vs*. high). MAC387^+^ tumor cells were divided into two groups according to the density of positive cells (0–2 *vs*. 3). Regression analyses were used to evaluate the association between the dichotomized markers and chemotherapy response. Mann-Whitney U test was used to evaluate the associations between continuous markers and response. Pearson Chi-Square or Fisher’s exact test was used for MAC387^+^ tumor cells (groups 0–3). The Kaplan-Meier method, log-rank testing, and Cox proportional hazards regression model were used in survival analyses. The survival time was calculated from the date of RC to the date of the last follow-up or death. All statistical tests were two-sided and p-values ≤ 0.05 were considered as statistically significant. The statistical analyses were performed with SPSS 21 (IBM).

## Electronic supplementary material

Dataset 1
