# Supplementary material for: Immunological tumor status may predict response to neoadjuvant chemotherapy and outcome after radical cystectomy in bladder cancer
Source: Sci Rep. 2017 Oct 4;7:12682. doi: 10.1038/s41598-017-12892-5 (PMC5627306; doi:10.1038/s41598-017-12892-5)
Supplement: Supplementary file 1 — Dataset 1 [file 41598_2017_12892_MOESM1_ESM.doc]

SUPPLEMENTARY MATERIAL

# ****Immunological tumor status may predict response to neoadjuvant chemotherapy and outcome after radical cystectomy in bladder cancer****

## Minna Tervahartiala^1^*, Pekka Taimen^2^, Tuomas Mirtti^3^, Ilmari Koskinen^4^, Thorsten Ecke^5^, Sirpa Jalkanen^1^, Peter J. Boström^6^

^1^ MediCity Research Laboratory, Department of Medical Microbiology and Immunology, University of Turku, Turku, Finland

^2^ Department of Pathology, University of Turku and Turku University Hospital, Turku, Finland

^3^ Helsinki University Hospital, Department of Pathology (HUSLAB) and Medicum, University of Helsinki

^4^ Department of Urology, Helsinki University Hospital and University of Helsinki, Helsinki, Finland

^5^ Department of Urology, HELIOS Hospital Bad Saarow, Bad Saarow, Germany

^6^ Department of Urology, Turku University Hospital, Turku, Finland

**Supplementary Table S1**. Marker counts from manually counted TMA samples and percentages from digitally counted whole sections.

|  | **Marker** | **n (%)** | **Mean/media (range)** |
| --- | --- | --- | --- |
| **TMA,**  **manual^1^** | **CD68** | 67 (99) | 5/1 (0-31) |
|  | **MAC387** | 67 (97) | 29/11 (0-188) |
|  | **CLEVER-1m^3^** | 66 (97) | 24/23 (0-66) |
|  | **CLEVER-1v^4^** | 67 (99) | 4/2 (0-34) |
| **Whole section,**  **digital^2^** | **CD68** | 57 (84) | 1.18/0.46 (0.006-9-17) |
|  | **MAC387** | 58 (85) | 4.51/4.28 (0.002-16.44) |
|  | **CLEVER-1m^3^** | 58 (85) | 4.73/3.76 (0.028-16.23) |
| ^1^ Marker counts from TMA, counted manually  ^2^ Positivity percentages from whole sections, counted with Fiji-ImageJ  ^3^ CLEVER-1 positive macrophages  ^4^ CLEVER-1 positive vessels | | | |

**Supplementary Table S2**. Correlations between manual and digital countings from whole sections, and TMA and whole sections (Spearman rank-order correlation coefficient).

| **Marker** | **Manual *vs.* digital**  **r_s_ (p)** | **TMA *vs.* whole sections**  **r_s_ (p)** |
| --- | --- | --- |
| **CD68** | 0.35 (0.008)* | 0.38 (0.002)* |
| **MAC387** | 0.65 (<0.001)* | 0.65 (<0.001)* |
| **CLEVER-1m^1^** | 0.51 (<0.001)* | 0.11 (0.41) |
| **CLEVER-1v^2^** |  | 0.10 (0.41) |
| ^1^ CLEVER-1 positive macrophages  ^2^ CLEVER-1 positive vessels  * Significant p-value | | |

**Supplementary Table S3**. Associations between clinicopathological characteristics and analysed markers.

| **Variable** | **CD68** | **MAC387** | **MAC387tumor^1^** | **CLEVER-1m^2^** | **CLEVER-1v^3^** |
| --- | --- | --- | --- | --- | --- |
| **Age** | -0.20 (0.11) | -0.025 (0.84) | 0.62 | 0.073 (0.56) | 0.10 (0.43) |
| **Gender** | 0.15 | 0.13 | 0.26 | 0.76 | 0.87 |
| **Smoking** | 0.22 | 0.85 | 0.55 | 0.25 | 0.36 |
| **cT category (TUR-BT’)** | 0.25 | 0.17 | 0.15 | 0.73 | 0.86 |
| **CIS^4^ (TUR-BT’)** | 0.19 | 0.41 | 0.71 | 0.93 | 0.57 |
| **LVI^5^ (TUR-BT’)** | 0.002* | 0.43 | 0.32 | 0.94 | 0.75 |
| **Tumor size (TUR-BT’)** | -0.28 (0.14) | -0.16 (0.44) | 0.40 | 0.037 (0.85) | -0.13 (0.52) |
| **pT category (RC”)** | 0.73 | 0.72 | 0.68 | 0.16 | 0.37 |
| **pN category (RC”)** | 0.73 | 0.18 | 0.71 | 0.60 | 0.29 |
| Spearman rank-order correlation coefficient, r_s_ (p), was used for age and tumor size (TUR-BT). Mann-Whitney U and Kruskal-Wallis test were used for other analyses (p).  ’ According to the TUR-BT pathology, imaging studies and clinical status  ” According to the pathological data from RC  ^1^ MAC387 positive tumor cells, semiquantitative scoring  ^2^ CLEVER-1 positive macrophages  ^3^ CLEVER-1 positive vessels  ^4^ Concomitant carcinoma in situ  ^5^ Lymphovascular invasion  * Significant p-value | | | | | |

**Supplementary Table S4**. Associations between neoadjuvant chemotherapy response and clinicopathological characteristics.

| **Variable** | **Complete**  ***vs.* other** | **Complete/Partial**  ***vs.* other** | **Progression**  ***vs.* other** |
| --- | --- | --- | --- |
| **Gender** | 1.00 | 0.17 | 0.11 |
| **Smoking** | 0.48 | 0.72 | 0.42 |
| **Neoadjuvant chemotherapy** | 0.64 | 0.63 | 0.26 |
| **CIS^1^ (TUR-BT’)** | 0.48 | 0.87 | 1.00 |
| **LVI^2^ (TUR-BT’)** | 1.00 | 0.77 | 0.75 |
| Pearson Chi-square/Fisher’s exact test.  ’ According to the TUR-BT pathology, imaging studies and clinical status  ^1^ Concomitant carcinoma in situ  ^2^ Lymphovascular invasion  ^3^ MAC387 positive tumor cells  ^4^ CLEVER-1 positive macrophages  ^5^ CLEVER-1 positive vessels  * Significant p-value | | | |

**Supplementary Table S5.** Dichotomized marker counts, n (%), in different neoadjuvant chemotherapy response groups.

| **Variable n (%)** | | **Complete response** | **Partial response** | **No response** | **Progression** |
| --- | --- | --- | --- | --- | --- |
| **CD68** | **Low** | 11 (37) | 8 (27) | 5 (17) | 6 (20) |
|  | **High** | 17 (45) | 6 (16) | 4 (11) | 11 (29) |
| **MAC387** | **Low** | 15 (41) | 8 (22) | 6 (16) | 8 (22) |
|  | **High** | 13 (42) | 6 (19) | 3 (10) | 9 (29) |
| **MAC387tumor^1^** | **Low** | 25 (47) | 11 (21) | 7 (13) | 10 (19) |
|  | **High** | 3 (20) | 3 (30) | 2 (13) | 7 (47) |
| **CLEVER-1m^2^** | **Low** | 16 (47) | 9 (27) | 2 (6) | 7 (21) |
|  | **High** | 12 (35) | 5 (15) | 7 (21) | 10 (29) |
| **CLEVER-1v^3^** | **Low** | 1x6 (41) | 7 (18) | 4 (10) | 12 (31) |
|  | **High** | 12 (41) | 7 (24) | 5 (17) | 5 (17) |
| Groups dichotomized according to the mean value.  ^1^MAC387 positive tumor cells; low (score 1-2), high (score 3)  ^2^ CLEVER-1 positive macrophages  ^3^ CLEVER-1 positive vessels | | | | | |
